# Administration of *Lactobacillus paracasei* HB89 mitigates PM_2.5_-induced enhancement of inflammation and allergic airway response in murine asthma model

**DOI:** 10.1371/journal.pone.0243062

**Published:** 2020-12-07

**Authors:** Ching-Hung Lin, Chia-Yi Tseng, Ming-Wei Chao

**Affiliations:** 1 Department of Bioscience Technology, Chung Yuan Christian University, Taoyuan, Taiwan; 2 Department of Biomedical Engineering, Chung Yuan Christian University, Taoyuan, Taiwan; 3 Center for Nanotechnology, Chung Yuan Christian University, Taoyuan, Taiwan; National Cheng Kung University, TAIWAN

## Abstract

PM_2.5_ causes abnormal immune response and asthma in animals. In this study, a Balb/c mouse animal model was exposed to PM_2.5_ to induce asthma. *Lactobacillus paracasei* HB89 was fed at the same time, in order to observe whether *L*. *paracasei* HB89 mitigates respiratory tract allergies stimulated by PM_2.5_. The results showed that PM_2.5_ stimulated a significant increase in white blood cells and immunoglobulin (IgE) in OVA-induced allergic Balb/c mice, and IgE in the blood further triggered the release of histamine in the lung immune cells. This not only increased overall immune cell counts, but the lymphocyte counts also increased significantly, resulting in significant inhibitions of cytokines INF-r and TGF-β, and induction of IL-4, IL-5, IL-13 and IL-17a. After feeding with HB89, apart from the absence of observable changes in body weight, the total white blood cell count in the animal blood and IgE response were also be reduced; the proliferation of immune cells in the lungs caused by PM_2.5_ was slowed down; and histamine and cytokines INF-r and TGF-β were secreted in large quantities, but IL- 4, IL-5, IL-13, IL-17a were inhibited, which effectively reduced the possibility of asthma induction.

## Introduction

In recent years, more and more attention has been paid to the impact of air pollution on our health. This has become a public health issue. Although the harm brought to any individual may seem insignificant, the harm caused to the global population must not be underestimated. Many epidemiological studies have shown that an increase in the concentration of air pollutants over short periods of time may increase the incidence rates of lung and cardiovascular diseases. Similarly, prolonged exposure to air pollutants results in increased overall death and incidence rates for cardiovascular diseases [1–5]. However, air pollutants consist of many kinds of substances. According to the US Environmental Protection Agency (EPA), the air pollutants that have the strongest impacts on our daily lives and health include NO_x_, SO_2_, O_3_, and PM_2.5_ (particles with a particle size of less than 2.5 μm) and PM_10_ (particles with a particle size of less than 10 μm) [6, 7]. These are currently considered the main components of air pollution{Tseng, 2016 #807}.

Previous literature has shown that suspended particles of different particle sizes distribute to different parts of the respiratory tract: PM_10_ deposits in the nasal cavity; PM_2.5_ enters the lungs; PM_0.1_ attaches to the pulmonary alveoli; and particles smaller than PM_0.1_ enter the blood stream via gas exchange [8–10]. Suspended particles will produce reactive oxygen species [11–14] after they enter the body, and form oxidative stress that leads to inflammatory response [13, 15–17]. Therefore, suspended particles may lead to inflammatory response due to oxidative stress [5]. This will indirectly affect many physical functions, such as immune function, as well as causing pathological changes in the respiratory system and neurodevelopmental disorders [18, 19], and reducing the efficiency of cardiac circulation [20]. Recent studies have shown that PM_2.5_ has a greater impact on children's respiratory diseases (such as asthma and allergies) than average adults [21, 22]. In addition, PM_2.5_ also increases the incidence and death rates of respiratory diseases in young children [23, 24]. Furthermore, exposure to PM_2.5_ may also cause chronic inflammation in pregnant women, causing congenital growth retardation in babies [25–28].

In order to increase physical immunity, products with *L*. *paracasei* added can protect against or help allergic patients change their states of health [29, 30]. An appropriate intake of *Lactobacillus* is an effective method. Many kinds of *Lactobacillus* on the market have similar effects. For example, the use of *L*. *paracasei* 33 products has been clinically proven to have an adjuvant effect on allergic rhinitis [31, 32]. There are clinical trials that have used *L*. *paracasei* GM080 for clinical atopic dermatitis [33]. *L*. *paracasei* in combination with *L*. *fermentum* has even been used to improve clinical symptoms of asthma in school-aged children [34]. Yogurt supplemented with *L*. *paracasei* N1115 probiotics is able to protect healthy elders from respiratory tract infections [35].

*L*. *paracasei* HB89 (BCRC910811) was isolated from the feces of healthy adults. The isolates have been included within functional development testing of *Lactobacillus paracasei*-33. It was identified via 16SDNA and API 50CHL as *L*. *paracasei* subsp. *paracasei*, a gram-positive bacteria that is without catalase oxidase or motility, is non-endospore-forming, and grows in both aerobic and anaerobic environments. Therefore, after inducing asthma in the animal model through exposure to PM_2.5_, and simultaneous feeding with *L*. *paracasei* HB89, we observed whether *L*. *paracasei* HB89 can mitigate respiratory tract allergies in environments stimulated by PM_2.5_.

## Materials and methods

### PM_2.5_ suspension

Particles (SKU-Pack Size: CRM558, Diesel—Clay Loam 1) were obtained from Sigma USA. Stock suspensions (20 mg/mL) of particles were prepared with the protocol described previously (Chao 2011) [5]. The particles powder (0.1 g) was suspended in 10 ml in PBS, 0.05% Tween-80 to make a 10 mg/ml DEP stock solution. Particles were then dispersed to achieve a particle size of PM2.5 (2.5 μm diameter and smaller) by vortexing for 3 min, then sonicating at 60 Hz for 5 min. Particle size was confirmed as PM_2.5_ using dynamic light scattering with the method shown in the previous publication.

### Animals

Female Balb/c mice (n = 6) aged 6 weeks were purchased from BioLASCO (Yilan, Taiwan) and Charles River Technology (Yilan, Taiwan), then acclimated for 14 days before the experiment was conducted. All animal experiments were approved by the Chung Yuan Christian University Institutional Animal Care and Use Committee (IACUC) with approved number 1071003. All mice were housed in the Chung Yuan Christian University vivarium, an IACUC and AAALAC approved facility, and supplied with standard food, water and daily inspection to ensure that they were not under pain or distress throughout the duration of the experiments. Animals were kept at 22 ± 1°C (mean ± SE), with 40–60% humidity and 12:12 h light-dark cycle with free access to laboratory chow and water throughout the study. All rodent surgical procedures, detail below, were carried out under full anesthesia utilizing a 90/10 mixture of ketamine/xylazine. Ketamine was administered at a dose of 100 mg/kg (Hospira Inc., Lake Forest, IL) and xylazine was administered at a dose of 10 mg/kg (AnaSed, Shenandoah, IA) by intraperitoneal (IP) injection. Depth of anesthesia was verified using the toe pinch method to ensure lack of reactivity and that rodents were fully anesthetized prior to commencement of any surgical procedures. The routine method of euthanasia for mice was inhalation of carbon dioxide under full anesthesia at the end of each experiment. Euthanasia of all mice was confirmed by physical examination to ensure heartbeat and respiration had ceased. This method of euthanasia is humane and rapid and is consistent with the recommendations of the Panel on Euthanasia of the Chung Yuan Christian University Animal Research Guidelines.

### Preparation of *Lactobacillus paracasei* HB89

HB9 was grown in MRS broth anaerobically at 37°C. The bacteria were incubated overnight and harvested via centrifuge at 3000 × g for 15 min at 4°C, before being resuspended in sterile phosphate buffer saline (PBS). Bacterial concentration and viability were determined via plate count. The HB89 fed mice were gavaged in 200 μL PBS (2×10^9^ CFU/mouse per day, respectively), using an aseptic gavage tube.

### Allergic sensitization, OVA challenge, and PM_2.5_ exposure

**Fig 1:**
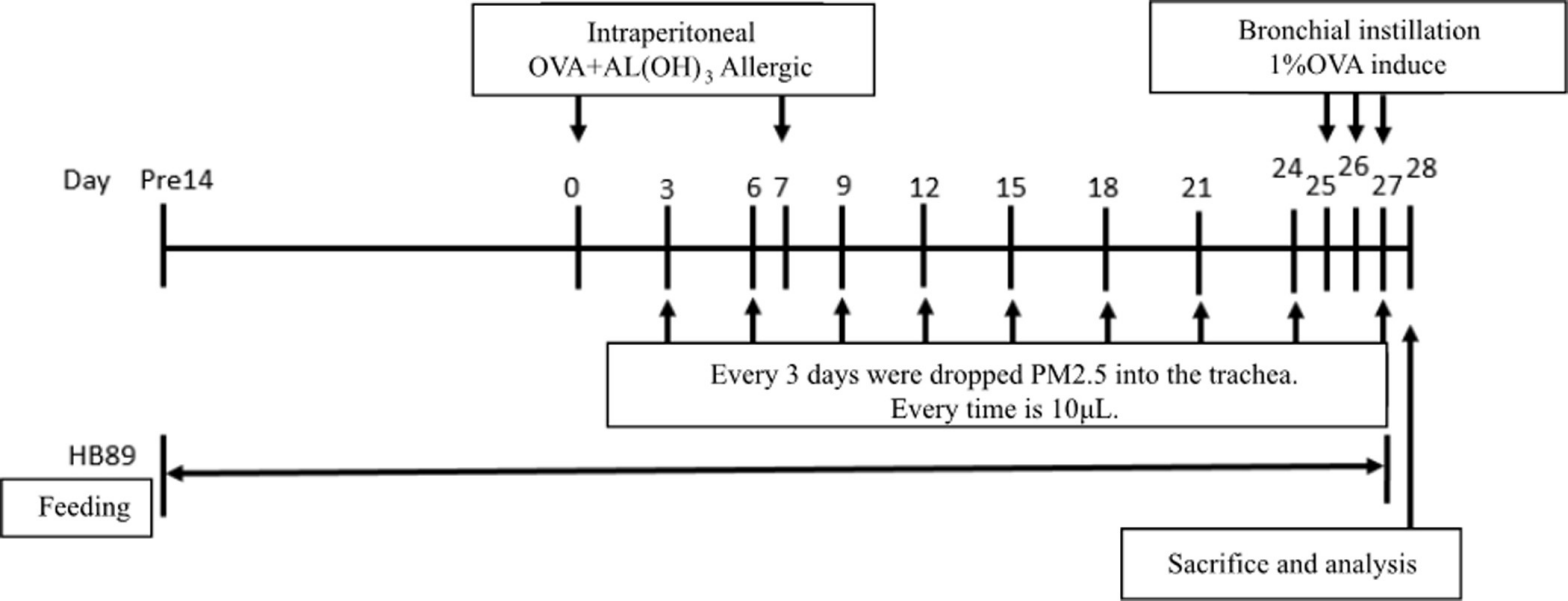
Experimental setup of the PM_2.5_ exposure enhanced mouse model of asthma.

Mice were assigned to one of five groups (n = 6/group): the PBS-sensitized and challenged control group (CON); the OVA-sensitized and challenged group (OVA); the PM_2.5_ extract exposed OVA group (OVA+PM_2.5_); the OVA-sensitized and challenged plus HB89 group; and the PM_2.5_ extract-exposed OVA plus HB89 group. All groups except for the CON group were intraperitoneally injected with 200 μL of aluminum hydroxide (Al(OH)_3_) and saline (1:1) containing 20 μg OVA (Sigma-Aldrich) on days 0 and 7, while the CON group received Al (OH)_3_ and saline as a control. On days 25, 26 and 27, the CON group and the other groups were challenged with either 50 μL saline or the same dose of OVA (20 μg/50 μL per mouse) via intranasal instillation. For OVA+PM_2.5_ and OVA+PM_2.5_+HB89 groups, mice were given an intratracheal instillation of 50 μL PM_2.5_ solution (100 μg/50 μL per mouse), over an interval of 3 days from day 3 to day 27. The instillation intratracheal protocols followed the previous study design [36]. At the same time, the same dose of control filter-extracted solution was intranasally administered to the OVA group. The control mice were sensitized and challenged with the same protocol using saline alone. All mice were anesthetized with isoflurane while being given intranasal instillation. The procedures for allergen sensitization and treatment are summarized in Fig 1.

### ELISA for serum cytokines and immunoglobulins

Blood was collected and serum was separated via centrifugation (5000 rpm, 4°C, 20 min) after resting at 4°C overnight. The supernatants were collected for cytokines (INF-r, IL-4, IL-5, IL-13, IL-17a, and TGF-b) and murine anti-OVA IgE analysis; the sediments suspended in PBS were used for a cell composition assessment. Serum was diluted 10-fold with 1X diluent buffer and aliquots of 100μL amniotic fluid were pipetted into black 96-well plates. All analyses and calibrations were performed in duplicate. Optical densities were determined using a commercial Cytokines and Anti-Ovalbumin IgE (mouse) ELISA Kit on an absorbance micro-reader (Promega, Madison, WI, USA) at 450 nm.

### Analysis of cell composition of bronchoalveolar lavage fluid

Bronchoalveolar lavage fluid (BALF) was collected from mice immediately following euthanasia by cervical dislocation, as previously described [24]. The BALF was placed on ice and centrifuged at 1500 rpm for 10 minutes at 4°C. The supernatants were collected for cytokines INF-r, IL-4, IL-5, IL-13, IL-17a, TGF-b, and histamine analysis and the sediments suspended with PBS were used for a cell composition assessment. Cell counts of macrophages, eosinophils, neutrophils and lymphocytes were performed by counting at least 200 cells in the suspended BALF, stained via Quick blood cell kit (Invitrogen) staining. The concentrations of cytokines and histamine were analyzed using a commercial Mouse ELISA Strip kit (Signosis, Inc., Sunnyvale, CA, USA) according to the manufacturer’s instructions. All analyses and calibrations were performed in duplicate. Optical densities were determined using an absorbance micro-reader (Promega, Madison, WI, USA) at 450 nm.

### Statistics

Data were expressed as mean values, presented as the mean ± SEM. The differences between experimental groups were analyzed using SPSS Statistics 20. Binary comparisons were made using the Student *t* test. Comparisons between multiple groups were assessed using a one-way ANOVA followed by a Kruskal-Wallis test. For all tests, *P*-values less than 0.05 were considered significant.

## Results

**Fig 2:**
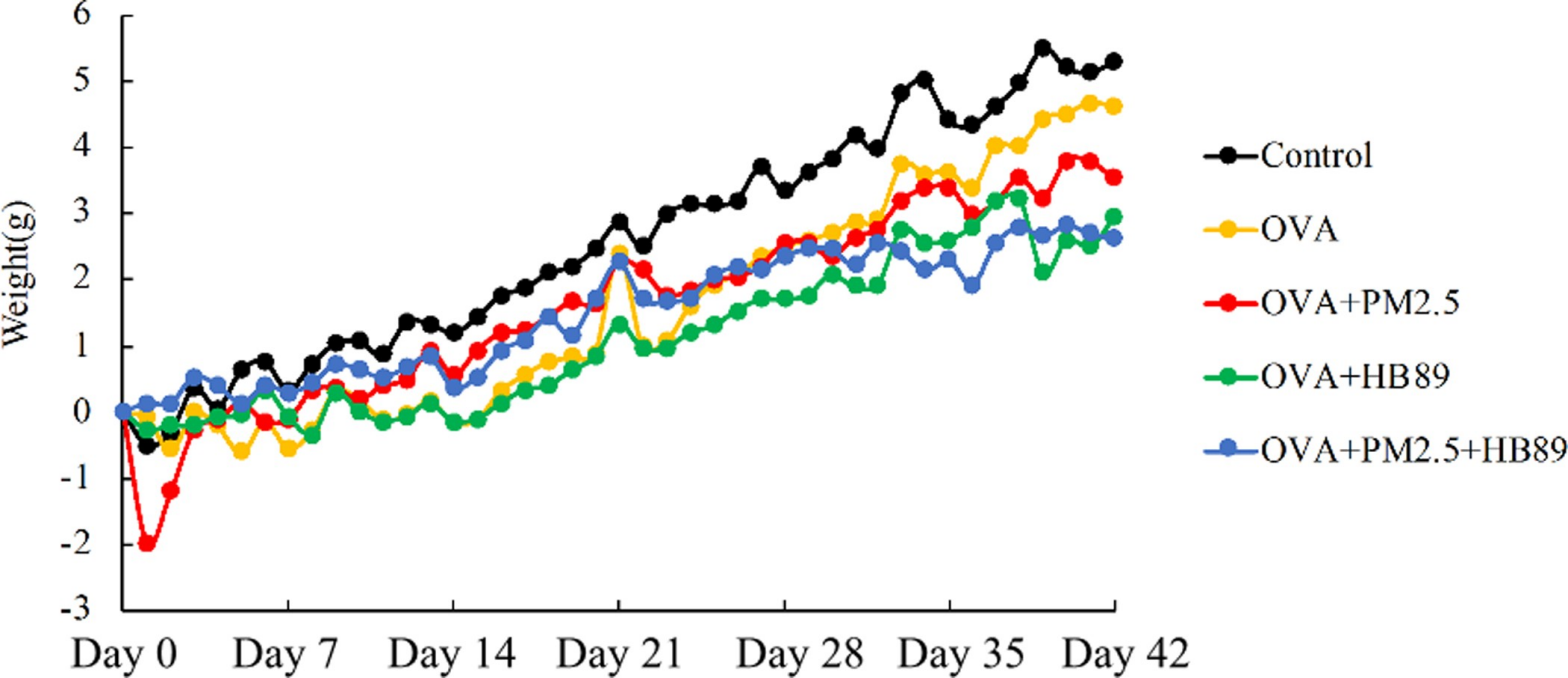
During the period from HB89 pre-feeding, 14 days before the allergy test, through to the allergy testing period (28 days), there was no difference in the weight of the Balb/c mice, other than weight gain over time.

**Fig 3:**
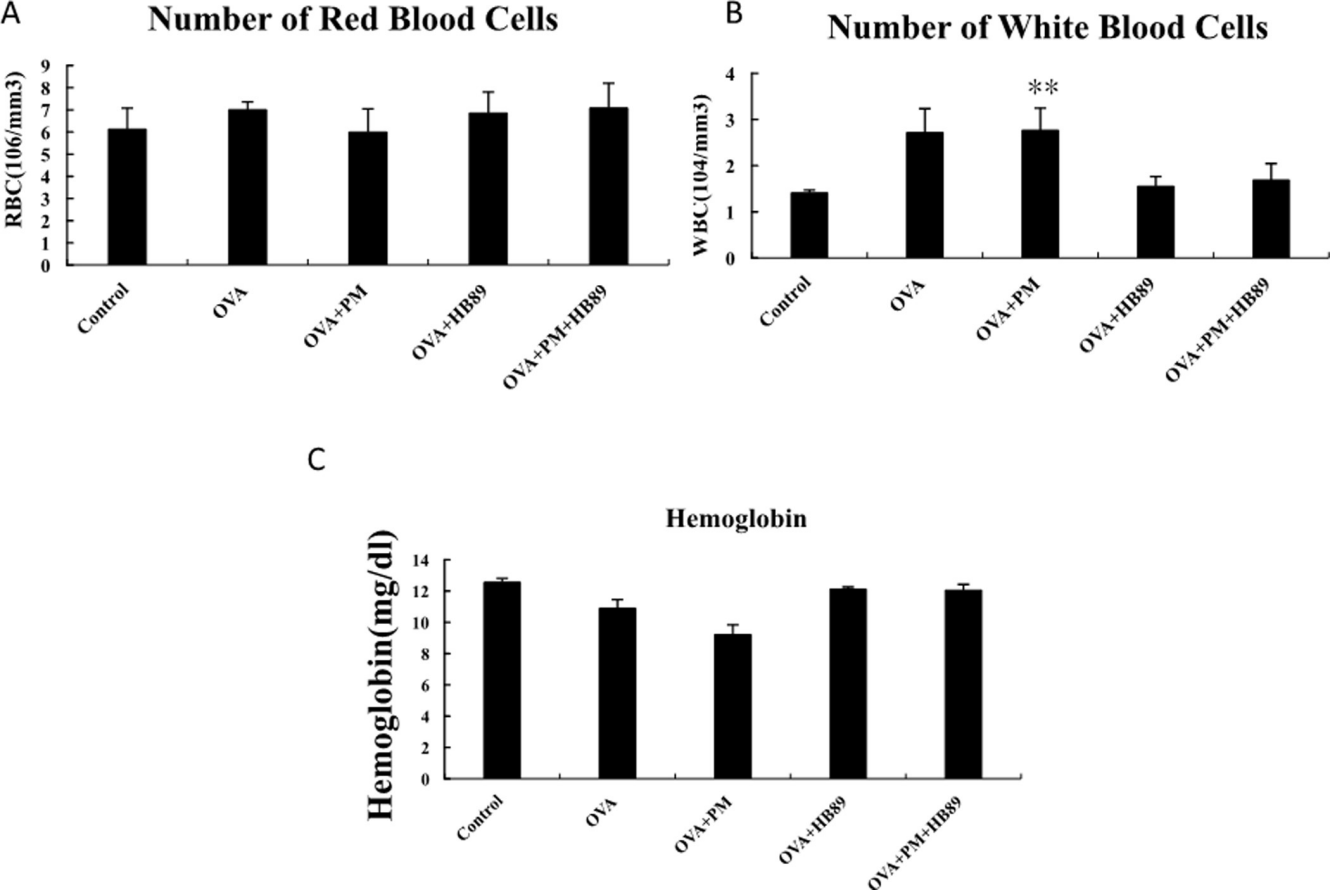
Differences in white blood cells, red blood cells, and hemoglobin in Balb/c mice: A. Red blood cells; B. White blood cells; C. Platelets; ** p <0.0l as compared with the control group.

Regarding the body weight comparison in Fig 2, weights in the control group were higher than those of other groups, but the difference was not significant. For the blood tests, the RBC counts (Fig 3A) were about 7–12.5 x 10^6^/mm^3^ in normal mice. With animals that had inhaled PM_2.5_, or which had OVA-induction or feeding with HB89, there was no significant difference in RBC count between groups. As shown in Fig 3B, the WBC count in normal mice was about 6–15 x 10^3^/mm^3^. The white WBC count was higher in the OVA-induced allergic group and the OVA+PM_2.5_ allergic group. The WBC counts in the control group, OVA allergic+*L*. *paracasei* HB89 group, and OVA+PM_2.5_ allergic + *L*. *paracasei* HB89 group all went down to a normal level, while the *L*. *paracasei* HB89 groups recorded a significant decrease in inflammatory response. Fig 3C shows the differences in hemoglobin. The hemoglobin value in normal mice is about 10.2–16.6 mg/dl; the hemoglobin values in the OVA+PM_2.5_ group were significantly lower, while the hemoglobin values in other groups remained normal.

**Fig 4:**
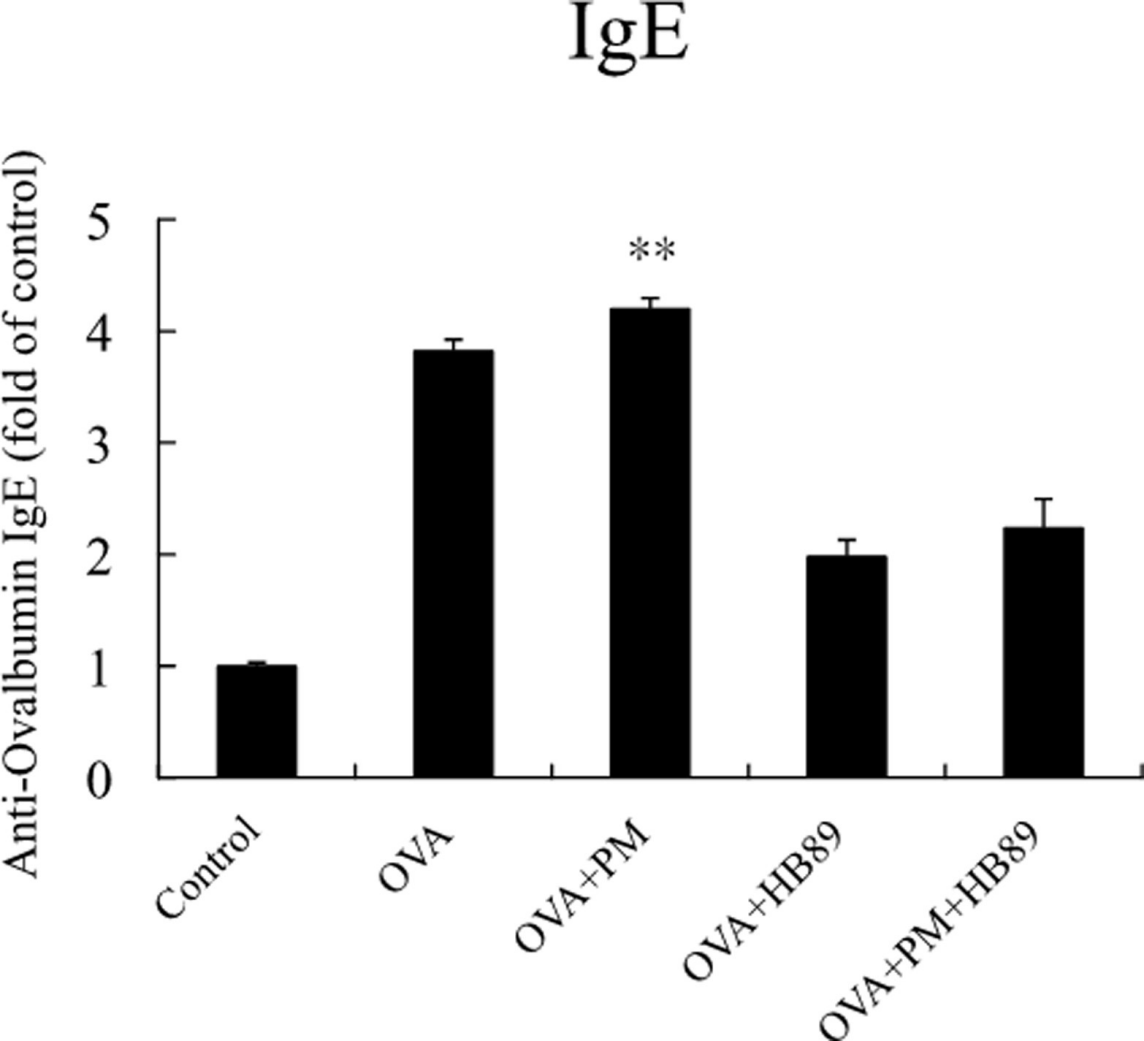
Differences in anti-OVA IgE concentration expressed in the serum of Balb/c mice; ** p <0.0l as compared with the control group.

**Fig 5:**
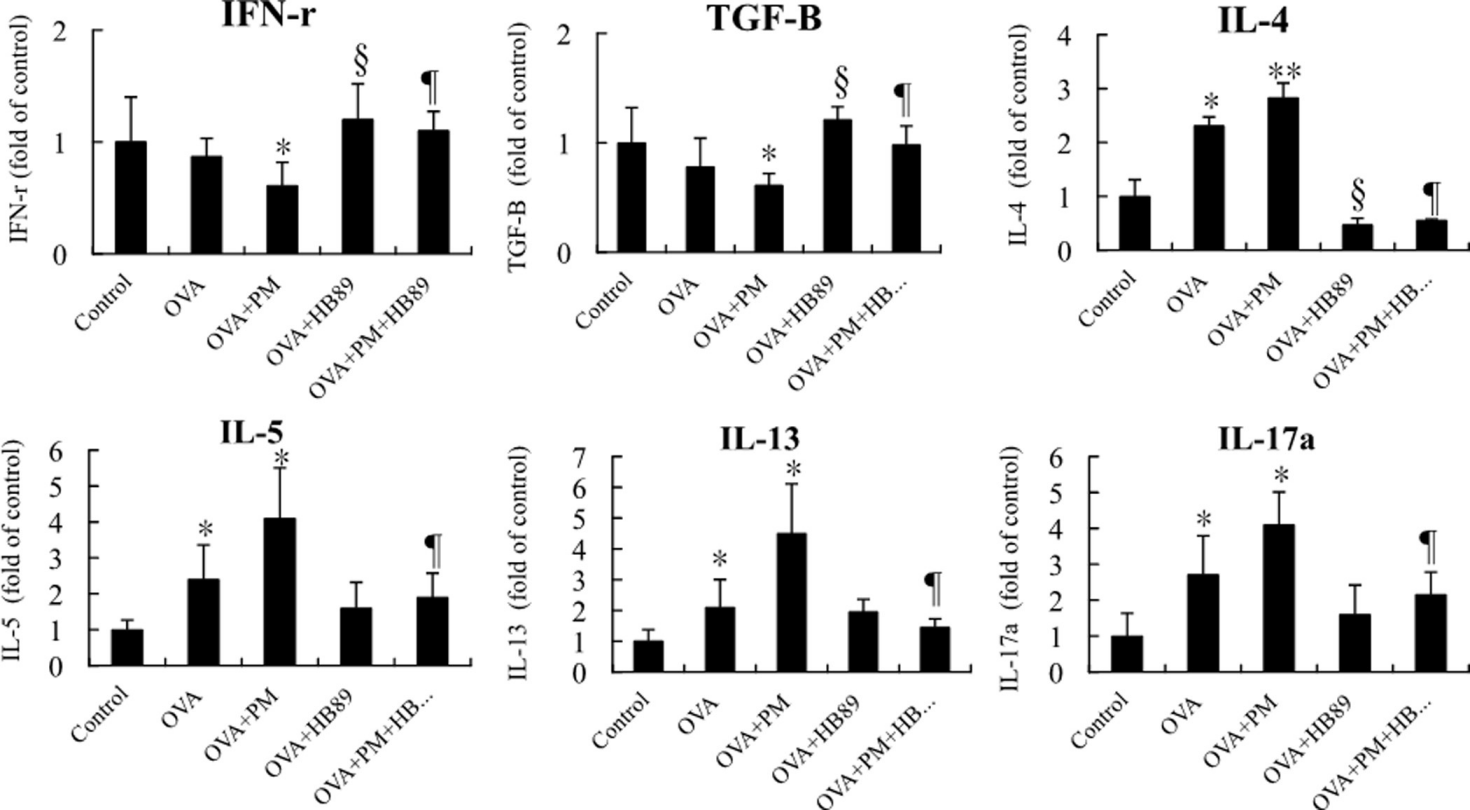
Counts of cytokines INF-r, TGF-β, IL-4, IL-5, IL-13 and IL-17a expressed in the serum of Balb/c mice; * and ** are p<0.05 and <0.0l as compared with the control group. § and ¶ are p<0.05 as compared with OVA and OVA+HB89 and OVA+PM and OVA+PM+HB89, respectively.

The concentration of immunoglobulin E (IgE) in the bloodstream is an important indicator for assessing allergic diseases and allergies (Fig 4). The IgE concentrations in the OVA allergic and OVA+PM_2.5_ allergic groups were about 3.5 to 4 times higher than the control group, while the group fed with *L*. *paracasei* HB89 had effectively reduced IgE concentrations. The immune system produces IgE in order to protect the body; IgE antibodies then remain in the body, and produce an allergic reaction the next time there is exposure to the same allergic substances. As a result, IgE concentrations are high in the bodies of those with allergic constitutions. Each kind of allergen can be matched with its specific IgE. Thus, from the results, it appears that the OVA-specific IgE concentration in the OVA-induced allergy groups remained high, whereas the group fed with *L*. *paracasei* HB89 had effectively reduced OVA-specific IgE concentrations. INF-r, IL-4, IL-5, IL-13, IL-17a, and TGF-β are cytokines that are expressed in large amounts when T cells, mast cells, basophils, and eosinophils are stimulated. As shown in Fig 5, immune cells will release these cytokines when there is inflammation in the body. INF-r and TGF-β were dropped with the induction of inflammation under OVA and OVA+PM_2.5_ exposure. So long as OVA-induced allergic groups are included, the IL-4, IL-5, IL-13, IL-17a released after stimulation are up to 3 times those of the control group, even reaching 4.5 times when stimulated with OVA+PM_2.5_ allergen. However, the group fed with *L*. *paracasei* HB89 had effectively reduced concentrations of OVA-induced or OVA+PM_2.5_ cytokines. Even IL-4 showed reductions to levels not significantly different from the control group.

**Fig 6:**
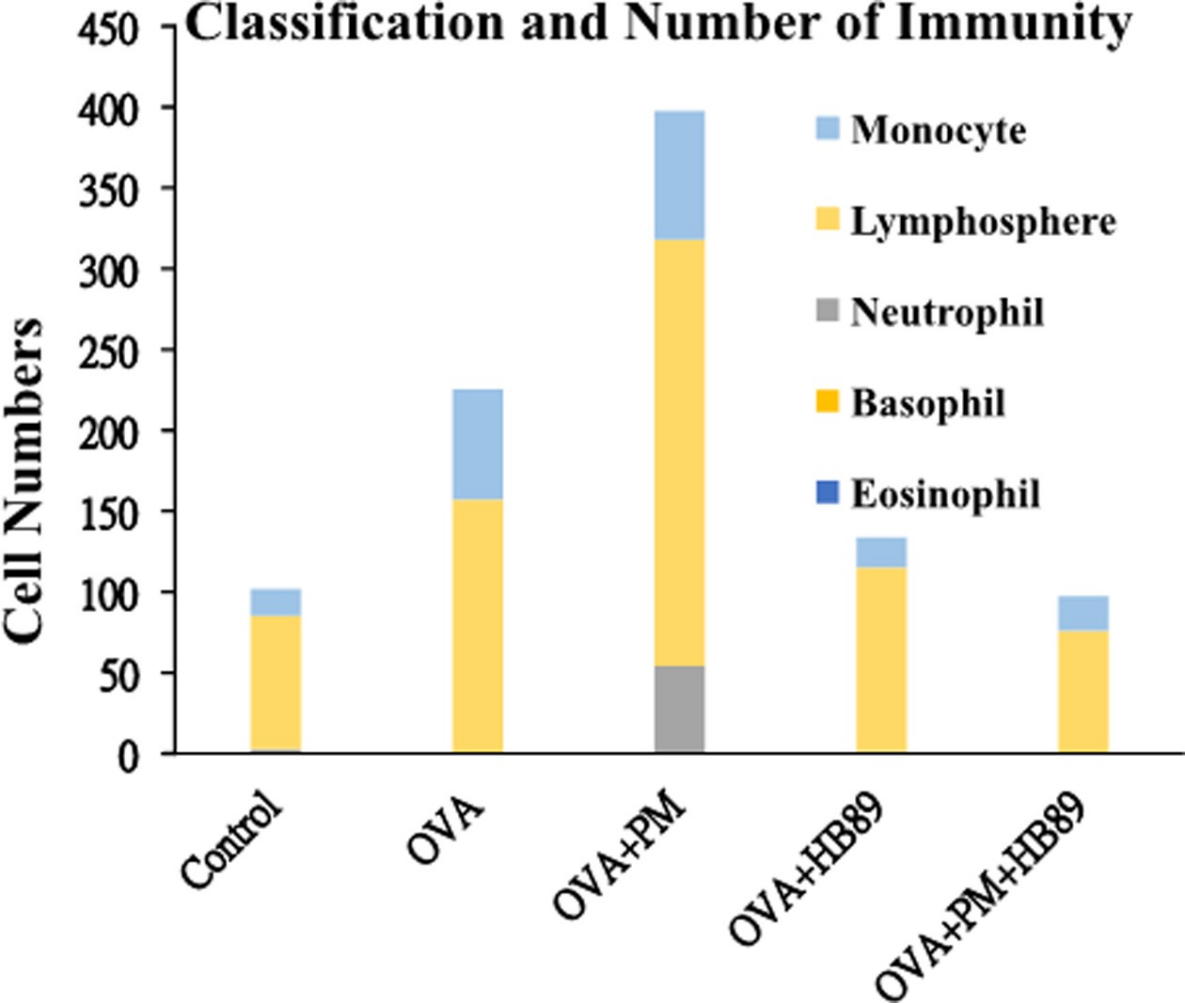
Analysis of immune cells in alveolar lavage fluid of Balb/c mice.

The immune system is activated when foreign substances invade. First, proliferation of immune cells is stimulated. As shown in Fig 6, the number of immune cells collected in the lung lavage fluid reached 2.25 times that of the control group when stimulated with OVA. However, when stimulated with OVA and PM_2.5_, the immune cell count increased to 3.98 times. The immune cell count decreased significantly—to the level of the control group—when fed with *L*. *paracasei* HB89. By analyzing the proportion of immune cells in the lung lavage fluid, we found that the lymphocyte counts in the OVA+PM_2.5_ allergic group increased to as much as c. 3.2 times those of the control group. The monocyte counts increased by 4.1 times in the OVA allergic group, and by 4.8 times in the OVA+PM_2.5_ allergic group. Neutrophils were detected in only small amounts in each group, but the OVA+PM_2.5_ allergic group was about 25 times higher than other groups. Eosinophils and basophils were found to have no difference from the control group. As the results showed, the total number of immune cells, lymphocytes and monocytes in the groups untreated with *L*. *paracasei* HB89 tended to be higher. This was especially true in the OVA+PM_2.5_ allergic group, where inflammatory response intensified by PM_2.5_ was seen, at counts up to c. 4 times higher. The group fed with *L*. *paracasei* HB89 had reduced inflammatory response, and the histamine results showed the same trend.

**Fig 7:**
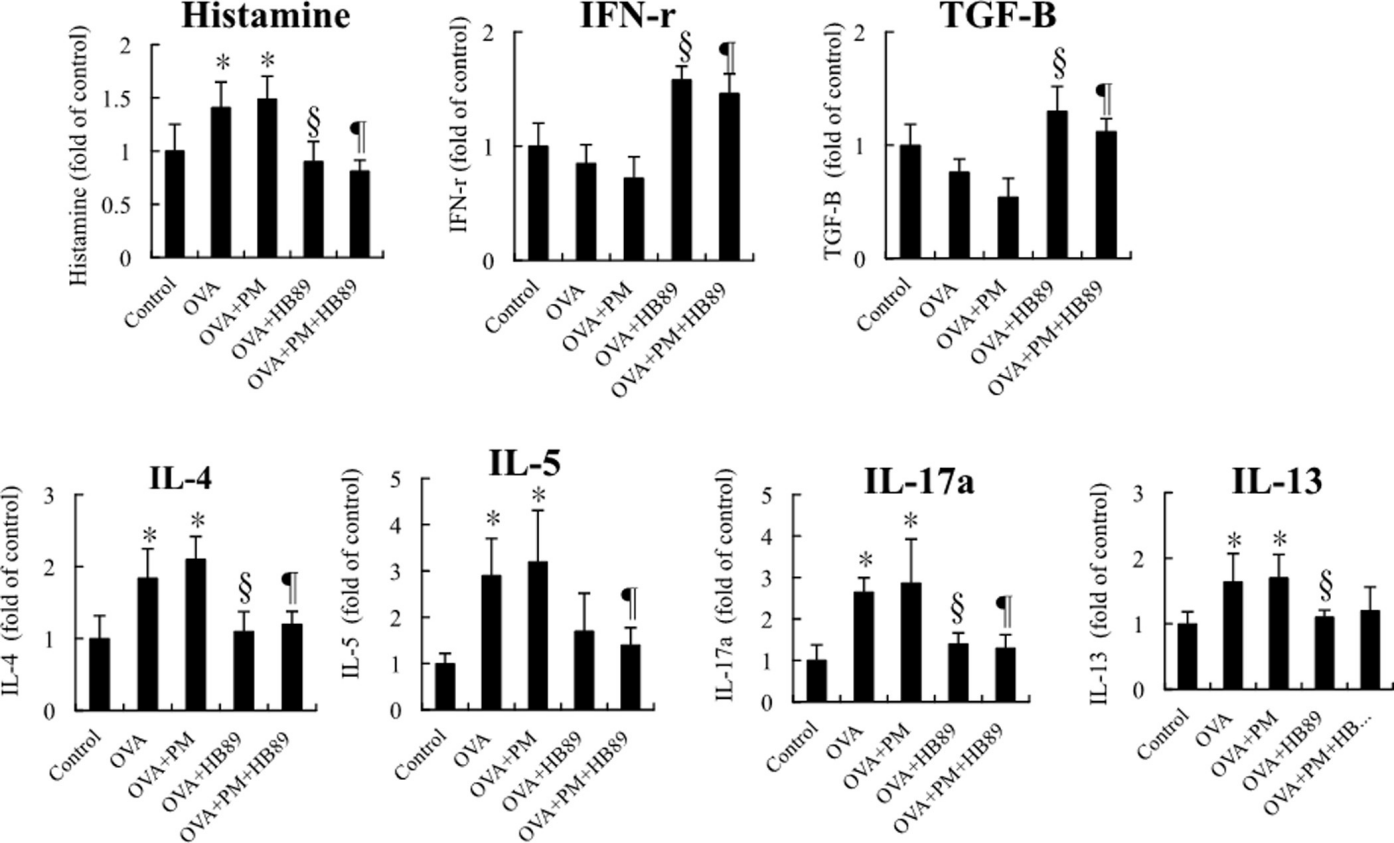
Analysis data for alveolar lavage fluid of Balb/c mice: A. Histamine; B. Differences in expression of cytokines INF-r, TGF-β, IL-4, IL-5, IL-13 and IL-17a. * and ** are p<0.05 and <0.0l as compared with the control group. § and ¶ are p<0.05 as compared with OVA and OVA+HB89 and OVA+PM and OVA+PM+HB89, respectively.

The immune system is activated when foreign substances invade the lungs, but excessive immune response causes allergic reactions, as shown with the IgE increases shown in Fig 3. However, once foreign substances came into contact with IgE, mast cells were triggered to release histamine (Fig 7). The concentration of histamine in the alveolar lavage fluid increased significantly, by c. 1.4 times, in both the OVA group and the OVA+PM_2.5_ group. However, after adding *L*. *paracasei* HB89, the expression of the OVA+*L*. *paracasei* HB89 and the OVA+PM_2.5_+*L*. *paracasei* HB89 groups showed no significant differences from the control group. However, INF-r and TGF-β were dropped with the induction of inflammation under OVA and OVA+PM_2.5_ exposure. IL-4, IL-5, IL-13, and IL-17a are cytokines expressed in large amounts when T cells, mast cells, basophils, and eosinophils are stimulated. The expression of these four cytokines was significantly lower in the OVA+*L*. *paracasei* HB89 group and the OVA+PM_2.5_+*L*. *paracasei* HB89 group than in the control group, while in the OVA and OVA+PM_2.5_ groups, it was several times higher than in the control group. Addition of *L*. *paracasei* HB89 to the diet significantly reduced the concentration of the cytokines IL-4, IL-5, IL-13, and IL-17a, showing a trend identical to those in Figs 4 and 5.

## Discussion

In a simulated allergic environment with individuals subjected to long-term chronic PM_2.5_ exposure, the frequency of exposure was much higher than the model in the Wang’s investigation. The stimulation was only via PM_2.5_ twice a day, at 4 hour intervals, the day before sacrificing; while Wang et al, 2016 is a study of acute PM_2.5_ stimulation, it still primarily uses OVA stimulation. In the present trial, PM_2.5_ was inhaled a total of 9 times, with subjects stimulated with PM_2.5_ every three days following trial initiation. This was primarily to simulate frequent inhalation of PM_2.5_ by allergic patients in realistic conditions, and to explore changes in blood, immune cells, and allergic indicators.

Chao et al., 2016 point out that exposure to PM_2.5_ reduces hemoglobin in the body, which is positively correlated with the occurrence of anemia. The present trial showed that hemoglobin also decreases within allergy+PM_2.5_ stimulation. Use of HB89 slows down the decrease of hemoglobin. This trial model focuses on daily preventive health care. Therefore, the daily feeding of animals with the HB89 strain, beginning 14 days before allergy induction, was different from the other group, where feeding with the HB89 strain only started on the sensitization day. The results of immune cell analysis showed that allergies combined with PM_2.5_ stimulation will indirectly trigger inflammatory response, and increased white blood cell counts. Inflammatory response was significantly slowed down in the OVA-HB89 and the OVA-PM_2.5_-HB89 groups.

Wang et al, 2016 is a report that contains no analysis of white blood cells in the bloodstream, primarily focusing on blood cell analysis results from respiratory tract lavage fluid; however, the results are significantly different from the cell results of this trial [37]. The results mostly correspond to those of wet allergic and inflammatory cells in allergic mice. For example, the number of eosinophils and neutrophils in the alveolar lavage fluid decreased after using HB89, and the macrophage counts increased after using HB89, but the lymphocyte counts did not differ significantly between groups. However, it was found in this trial that with long-term chronic PM_2.5_ stimulation, the lymphocyte counts in the OVA-PM_2.5_ group were approximately 3.2 times as high as those in the control group. Monocyte counts increased in the OVA group, and increased 4.8 times with PM_2.5_ inhalation (OVA-PM_2.5_). In the groups that used HB89, monocyte counts decreased. Only small neutrophil counts were found in any of the groups, but the OVA-PM_2.5_ group showed 25 times higher than the other groups. Differences were found in both eosinophils and basophils. The results showed that the total counts of immune cells, lymphocytes, and monocytes tended to be higher in the groups without *L*. *paracasei* HB89 treatment. This was especially true with the OVA+PM_2.5_ allergic group, where inflammatory response intensified by PM_2.5_ was seen, at up to 4 times greater levels. The group fed with *L*. *paracasei* HB89 had reduced inflammatory responses, and the histamine results shared the same trend. In 2018, Jiaxiang Zhang, et al. Zhang et al, 2019 investigated immune cell changes in alveolar lavage fluid from allergic mice exposed to PM_2.5_ [38]. The trial model was to apply stimulation with PM_2.5_ continuously three days before sacrificing. The results showed an increase in the total immune cell counts in the allergy+PM_2.5_ group’s alveolar lavage fluid (results similar to those of this trial), and a significant increase in lymphocyte and eosinophil counts. That study found that PM_2.5_ may intensify asthma by intensifying M2 macrophages [39]. This is the first time that eotaxin-1 released by M2 macrophages was found to play a vital role in the pathogenesis of asthma [38]. The distribution of monocytes (monocytes being the predecessors of macrophages) in the present trial was essentially similar to the results of that prior trial. Increases in the frequency of PM_2.5_ stimulation change the way the body's immune system functions. The above results illustrate the differences in test models, and how differences in test materials make a difference in trial results.

Therefore, this may mean that HB89 can mitigate allergic reactions of allergy patients undergoing acute stimulation with PM_2.5_ (short-term exposure) (no studies or reports related to chronic long-term exposure to PM_2.5_ have at present been found); and that HB89 can improve the lymphocyte and monocyte counts, as well as histamine concentrations, within the immune cells of allergic subjects chronically exposed to PM_2.5_. HB89 can mitigate acute stimulation, while HB89 can mitigate chronic long-term exposure. In conclusion, these results confirm that long-term consumption of *L*. *paracasei* HB89 products can effectively mitigate allergic response and reduce inflammatory response caused by PM_2.5_.
